# The emerging neurobiology of calorie addiction

**DOI:** 10.7554/eLife.01928

**Published:** 2014-01-07

**Authors:** Cristina García-Cáceres, Matthias H Tschöp

**Affiliations:** 1 **Cristina García-Cáceres** is at the Helmholtz Diabetes Center, Helmholtz Zentrum München, Munich, Germanygarcia-caceres@helmholtz-muenchen.de; 2 **Matthias H Tschöp** is at the Helmholtz Diabetes Center, Helmholtz Zentrum München, Munich, Germany and Division of Metabolic Diseases, Technische Universität München, Munich, Germanytschoep@helmholtz-muenchen.de

**Keywords:** obesity, metabolism, nutrient, feeding behavior, optogenetics, neuronal circuits, Mouse

## Abstract

The response of the brain to sugar is determined by specific cell populations in the brain, including neurons that secrete melanin-concentrating hormone, and culminates in the release of dopamine.

**Related research article** Domingos AI, Sordillo A, Dietrich MO, Liu Z-W, Tellez LA, Vaynshteyn J, Ferreira JG, Ekstrand MI, Horvath TL, de Araujo IE, Friedman JM. 2013. Hypothalamic melanin concentrating hormone neurons communicate the nutrient value of sugar. *eLife* **2**:e01462. doi: 10.7554/eLife.01462

**Image** Optogenetic control of MCH neurons has shed new light on how the brain responds to sucrose; scale bar is 15 µm

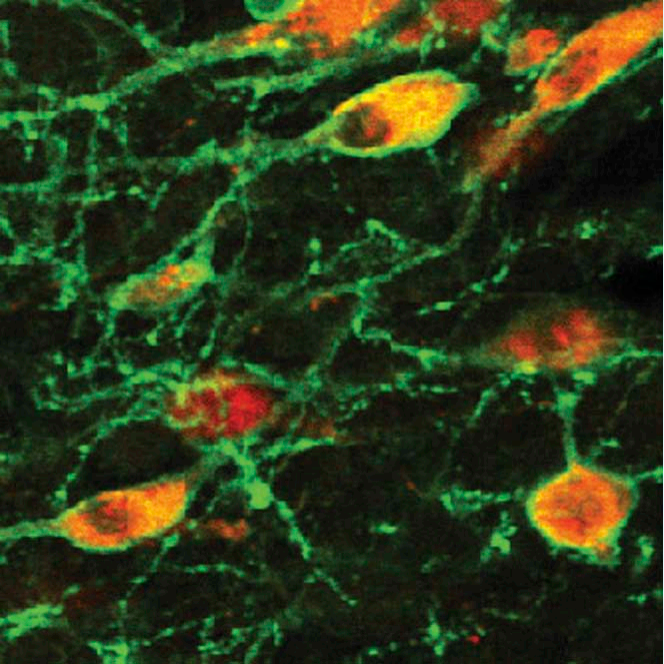

The increased availability and consumption of highly palatable foods is the major factor behind the rise of obesity and type 2 diabetes in developed countries. Many of these highly palatable foods are sweet tasting foods that contain high levels of the natural sugar sucrose. There are alternatives to sucrose—the artificial sweetener sucralose is 600 times sweeter and does not contain calories—but the obesity epidemic continues because we prefer sucrose to sucralose.

Progress has been made in recent years in understanding the neurobiological underpinnings for this preference: sucrose activates dopamine neurons in a region of the brain called the striatum, and the resulting release of dopamine is associated with pleasure (Velloso et al., 2008). Sucralose, on the other hand, does not have this effect. Moreover, the repeated consumption of high levels of sucrose can create a cycle of continued overconsumption—even compulsive eating—in order to recapture the initial feelings of pleasure. This is similar in many ways to drug abuse or addiction, and also involves some of the same signalling pathways within the body (Johnson and Kenny, 2010).

In 2011 Jeff Friedman of Rockefeller University and co-workers—including Ana Domingos as first author—used optogenetic techniques to show that activating dopamine neurons in the brain encouraged mice to eat in the absence of a sweet taste (Domingos et al., 2011). The mice preferred water to sugar if their dopamine neurons were activated at the same time that they were offered the water.

Now, in *eLife*, a team of researchers led by Domingos and Friedman reports that the release of dopamine is driven by melanin-concentrating hormone (MCH) neurons in the lateral hypothalamus of the brain (Domingos et al, 2013a). The experiments relied on transgenic mice in which the MCH neurons could be activated with a carefully directed light stimulus, and showed that mice preferred sucralose to sucrose when their MCH neurons had been activated. This finding was confirmed with mutant mice that lacked the cellular machinery required to recognize sweet tastes; these mice still preferred sucrose over sucralose because of its caloric content.

Friedman, Domingos (who is now at the Gulbenkian Science Institute in Portugal) and co-workers—who are based at Rockefeller, Yale, Albert Einstein College of Medicine, the JB Pierce Laboratory and UFRGS in Brazil—then engineered mice that did not have any MCH neurons at all. The fact that these mice preferred sucralose to sucrose supports a model in which MCH neurons appear to function as both a sucrose sensor and a nutrient sensor. Taken together, the results demonstrate that preference for sucrose is dependent on at least three factors: its ability to activate MCH neurons; its sweet taste as detected by the taste buds; and, as previously reported, the metabolic state of the organism. (Domingos et al., 2011; Domingos et al., 2013b). Consistent with these findings, they propose a model in which MCH neurons act as a key component of the neuronal networks that lead to the release of dopamine in response to the detection of sucrose (Figure 1).

**Figure 1.**
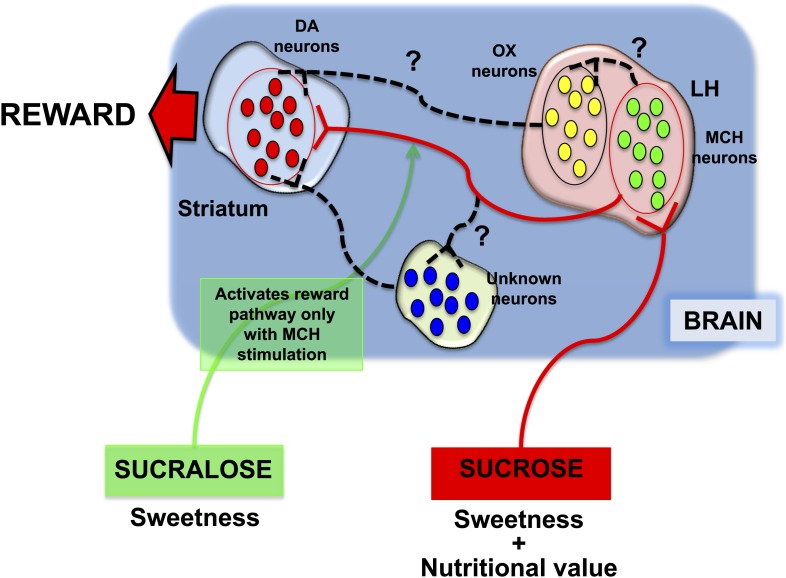
Different responses to sucrose and sucralose. Domingos et al. showed that sucrose activates melanin-concentrating hormone (MCH) neurons in the lateral hypothalamus (LH), which leads to the release of dopamine (DA) in the striatum: pathway shown in red. In contrast, sucralose can only induce the release of dopamine if optogenetic techniques are used to activate the MCH neurons; pathway shown in green. However, it is almost certain that various other neuronal pathways (shown by black dashed lines) are involved; these might include orexin/hypocretin (OX) neurons in the lateral hypothalamus, or other neurons elsewhere in the brain.

While this latest work shows that sucrose preference is based on its nutritive value, the actual molecular mechanisms via which it affects MCH neurons remains unknown. According to one report, since sucrose contains glucose, it could modulate the excitability of MCH neurons via potassium channels that are gated by ATP (Kong et al., 2010).

Domingos et al. now suggest that the activation of the MCH neurons by sucrose might be mediated indirectly by afferent nerve fibres originating from taste sensors in the taste buds and/or the gastrointestinal tract. The receptors that respond to sweet tastes also respond to the nutritional content of the food and help determine which neuropeptides should be released by taste buds (Geraedts and Munger, 2013). Moreover, sucrose, unlike sucralose, is known to act on taste receptors in the gastrointestinal tract (Steinert et al., 2011): this modulates the release of hormones which serve to inform the brain about the availability of calories in the body (Brown et al., 2011). Taken together, these data suggest that taste receptors in both the gustatory system (the part of the sensory system that responds to taste) and the gastrointestinal system may respond to natural sugars in one way, and to artificial sugars in a different way.

An outstanding challenge is to find out how MCH neurons activate dopamine neurons in the striatum. One possibility is that the orexin/hypocretin neurons in the hypothalamus have a role. Since these neurons are involved in the regulation of the desire for sugar (Cason and Aston-Jones, 2013), and they are also known to interact with MCH neurons (Guan et al., 2002), they might work together with MCH neurons to trigger reward.

The work of Domingos et al. represents a significant advance in our understanding of the neural mechanisms underlying how mammals respond to some kinds of foods. We now understand better why we have an innate desire for sweet foods, which are highly caloric and might have been, in the past, advantageous from an evolutionary perspective. Yet in the modern world, where highly caloric food is readily available, how do we resist this drive so as to avoid the many problems that are associated with obesity?

Interestingly, it appears that the increased use of non-caloric sugar substitutes as a mechanism to prevent weight gain or enhance weight loss has come at a cost. Recent studies show that prolonged consumption of sucralose and other high-intensity sweeteners can have potentially harmful effects on energy metabolism (Swithers et al., 2013; Simon et al., 2013). On the other hand, it has been reported that non-caloric sugar substitutes do little to reduce feelings of hunger (Brown et al., 2011).

By applying a combination of novel technologies to a question that nutrition researchers have been investigating for many years, Domingos et al. have discovered that a specific subset of neurons in the hypothalamus are essential for both calorie sensing and triggering the reward value associated with sucrose. A great deal of work is required to understand how this circuit relates to higher brain centres and other known nutrient-sensing cells. But thanks to the elegant studies by Domingos, Friedman and their co-workers, a door to that path has just been pushed wide open.

## References

[bib1] Brown AW Bohan Brown MM Onken KL Beitz DC 2011 Short-term consumption of sucralose, a nonnutritive sweetener, is similar to water with regard to select markers of hunger signaling and short-term glucose homeostasis in women. Nutrition Research 31:882–888. 10.1016/j.nutres.2011.10.004 22153513

[bib2] Cason AM Aston-Jones G 2013 Role of orexin/hypocretin in conditioned sucrose-seeking in rats. Psychopharmacology 226:155–165. 10.1007/s00213-012-2902-y 23096770 PMC3572270

[bib3] Domingos A Vaynshteyn J Voss HU Ren X Gradinaru V Zang F Deisseroth K de Araujo IE Friedman J 2011 Leptin regulates the reward value of nutrient. Nature Neuroscience 14:1562–1568. 10.1038/nn.2977 PMC4238286 22081158

[bib4] Domingos AI Sordillo A Dietrich MO Liu Z-W Tellez LA Vaynshteyn J Ferreira JG Ekstrand MI Horvath TL de Araujo IE Friedman JM 2013a Hypothalamic melanin concentrating hormone neurons communicate the nutrient value of sugar. eLife 2:e01462. 10.7554/eLife.01462 24381247 PMC3875383

[bib5] Domingos A Vaynshteyn J Sordillo A Friedman J 2013b The reward value of sucrose in leptin deficient obese mice. Molecular Metabolism. 10.1016/j.molmet.2013.10.007 PMC3929919 24567906

[bib6] Geraedts MC Munger SD 2013 Gustatory stimuli representing different perceptual qualities elicit distinct patterns of neuropeptide secretion from taste buds. Journal of Neuroscience 33:7559–7564. 10.1523/JNEUROSCI.0372-13.2013 23616560 PMC3687534

[bib7] Guan JL Uehara K Lu S Wang QP Funahashi H Sakurai T Yanagizawa M Shioda S 2002 Reciprocal synaptic relationships between orexin- and melanin-concentrating hormone-containing neurons in the rat lateral hypothalamus: a novel circuit implicated in feeding regulation. International Journal of Obesity 26:1523–1532. 10.1038/sj.ijo.0802155 12461668

[bib8] Johnson PM Kenny PJ 2010 Dopamine D2 receptors in addiction-like reward dysfunction and compulsive eating in obese rats. Nature Neuroscience 13:635–641. 10.1038/nn.2519 PMC2947358 20348917

[bib9] Kong D Vong L Parton LE Ye C Tong Q Hu X Choi B Bruning JC Lowell BB 2010 Glucose stimulation of hypothalamic MCH neurons involves K(ATP) channels, is modulated by UCP2, and regulates peripheral glucose homeostasis. Cell Metabolism 12:545–552. 10.1016/j.cmet.2010.09.013 21035764 PMC2998191

[bib10] Simon BR Parlee SD Learman BS Mori H Scheller EL Cawthorn WP Ning X Gallagher K Tyrberg B Assadi-Porter FM Evans CR MacDougald OA 2013 Artificial sweeteners stimulate adipogenesis and suppress lipolysis independently of sweet taste receptors. Journal of Biological Chemistry 288:32475–32489. 10.1074/jbc.M113.514034 24068707 PMC3820882

[bib11] Steinert RE Frey F Topfer A Drewe J Beglinger C 2011 Effects of carbohydrate sugars and artificial sweeteners on appetite and the secretion of gastrointestinal satiety peptides. British Journal of Nutrition 105:1320–1328. 10.1017/S000711451000512X 21255472

[bib12] Swithers SE Sample CH Davidson TL 2013 Adverse effects of high-intensity sweeteners on energy intake and weight control in male and obesity-prone female rats. Behavioral Neuroscience 127:262–274. 10.1037/a0031717 23398432 PMC3985091

[bib13] Velloso LA Araujo EP de Souza CT 2008 Diet-induced inflammation of the hypothalamus in obesity. Neuroimmunomodulation 15:189–193. 10.1159/000153423 18781083

